# An Absence of Statesmanship in B.M.A. Policy

**Published:** 1918-11-30

**Authors:** 


### AN ABSENCE OF STATESMANSHIP IN B.M.A. POLICY.

THE *British Medical Journal* of November 23 devotes space to a criticism of the article on "A State Medical Service" (see THE HOSPITAL for November 9 and 16). It is matter for regret that there is not one word of constructive criticism in the whole two columns of the leader. None who has read Colonel Maurice's article can fail to be struck by the restraint and common sense that characterise what is the first published attempt to outline an organisation for a State Medical Service. There is not a dogmatic sentence in the article, nor is there any advocacy. It is only a suggestion of what a State Medical Service might be. The greatest care is taken to suggest measures that shall enable good men to reap reward for their work, and that shall stimulate all but the hopelessly average to improvement and exertion. We are not deeply enamoured of the idea of State Medical Service, but we cannot but be aware that the signs of the times point that way, and therefore we welcomed the opportunity to put before our readers a carefully considered scheme. If we believed those who control it, and that the *British Medical Journal* really represented all the members of the British Medical Association, we might conclude such a criticism as this one was the best the Association could produce. In that case impartial observers might despair of the future of the medical profession, but we incline to the belief that the majority of the Association is more alive to the exigencies of the times than are those who profess to lead it. The rank and file of the Association may yet offer proposals and schemes for a State Medical Service with constructive criticism of that set forth in our columns. Colonel Maurice has warned the medical profession against a *non possumus* attitude, and has called for statesmanship. Statesmanship appears to be at a premium with the head of the British Medical Association, but there should be plenty of it amongst its members, and we hope they may become articulate.

